# An autopsy case of infective aortic aneurysm with *Pasteurella multocida* infection: clinicopathological appearance and a review of literatures

**DOI:** 10.1186/s12941-023-00611-0

**Published:** 2023-07-11

**Authors:** Kazuhiro Nomoto, Yukiko Hata, Shojiro Ichimata, Syu Mizuno, Naoki Nishida

**Affiliations:** 1 Department of Pathology, Koseiren Takaoka Hospital, 5-10 Eiraku-cho, Takaoka, Toyama 933-8555 Japan; 2 Department of Legal Medicine, Faculty of Medicine, University of Toyama, 2630 Sugitani, Toyama, Toyama 930-0194 Japan; 3 Division of General Medicine and Infectious Diseases, Koseiren Takaoka Hospital, 5-10 Eiraku-cho, Takaoka, Toyama 933-8555 Japan

**Keywords:** Autopsy, Infective aortic aneurysm, *Pasteurella multocida*, Sepsis

## Abstract

Here, we showed our clinicopathological findings of infected aortic aneurysm (IAA) with *Pasteurella multocida*, which is a Gram-negative coccobacillus and is part of the normal oral flora of many animals. The patient was a 76-year-old male animal owner with a history of diabetes mellitus, alcoholic liver damage, and laryngeal cancer. He died 16 days after admission without undergoing operation because of poor general condition. Autopsy showed saccular outpouching with loss of the existing aortic wall and marked neutrophilic infiltration in the suprarenal abdominal aorta. Rupture was not evident. A polymerase chain reaction assay using DNA extracted from formalin-fixed paraffin-embedded specimen of the aneurysmal wall detected the *Pasteurella multocida* gene, therefore we conclude that the present case was IAA of native aorta with *Pasteurella multocida* infection. A review of the literature showed that IAA of native aorta with *Pasteurella multocida* infection is opportunistic and that liver disorder, alcohol addiction, diabetes mellitus, and animal bite may increase its risk. On the other hand, aortic endograft infection with *Pasteurella multocida* frequently occurred without an immunocompromised state. *Pasteurella multocida* may be a distinct causative microorganism in IAA, and/or sepsis when the participant is an animal owner.

**Supplementary Information:**

The online version contains supplementary material available at 10.1186/s12941-023-00611-0.

## Introduction

Infected aortic aneurysm (IAA), also known as mycotic aneurysm, is a rare complication of bacterial or fungal infection, and its prevalence is less than 2% of all aortic aneurysms in Europe and the USA and reaches as high as 13% in Taiwan [1]. Many cases are complicated with uncontrolled sepsis and/or rupture of the wall leading to fatal hemorrhage [2]. In a recent clinical study of 182 IAA cases, 128 cases (70.3%) had positive blood and/or tissue culture: *Staphylococcus aureus* was found in 38 cases (20.9%), *Streptococcus* sp. in 37 cases (20.3%), *Salmonella* sp. in 19 cases (10.4%), *Enterococcus* sp. in 16 cases (8.8%), Gram-negative intestinal bacteria in six cases (3.3%) other species in 12 cases (6.6%), and 54 cases (29.7%) had negative culture [2].

Here, we described the clinicopathological appearance of the extremely rare autopsy cases with IAA due to *Pasteurella multocida* (*P. multocida*), which is a Gram-negative coccobacillus and is part of the normal oral flora of many animals, including cats, dogs, and rabbits [3].

## Case description

A 76-year-old man was transferred to the hospital by ambulance due to shaking rigor with mild consciousness disturbance. The patient experienced loss of appetite for 1 month and had a history of hypertension, diabetes mellitus, alcoholic liver damage, and hypothyroidism. Furthermore, the patient was scheduled to be admitted to the hospital under the diagnosis of advanced laryngeal cancer.

**Fig. 1:**
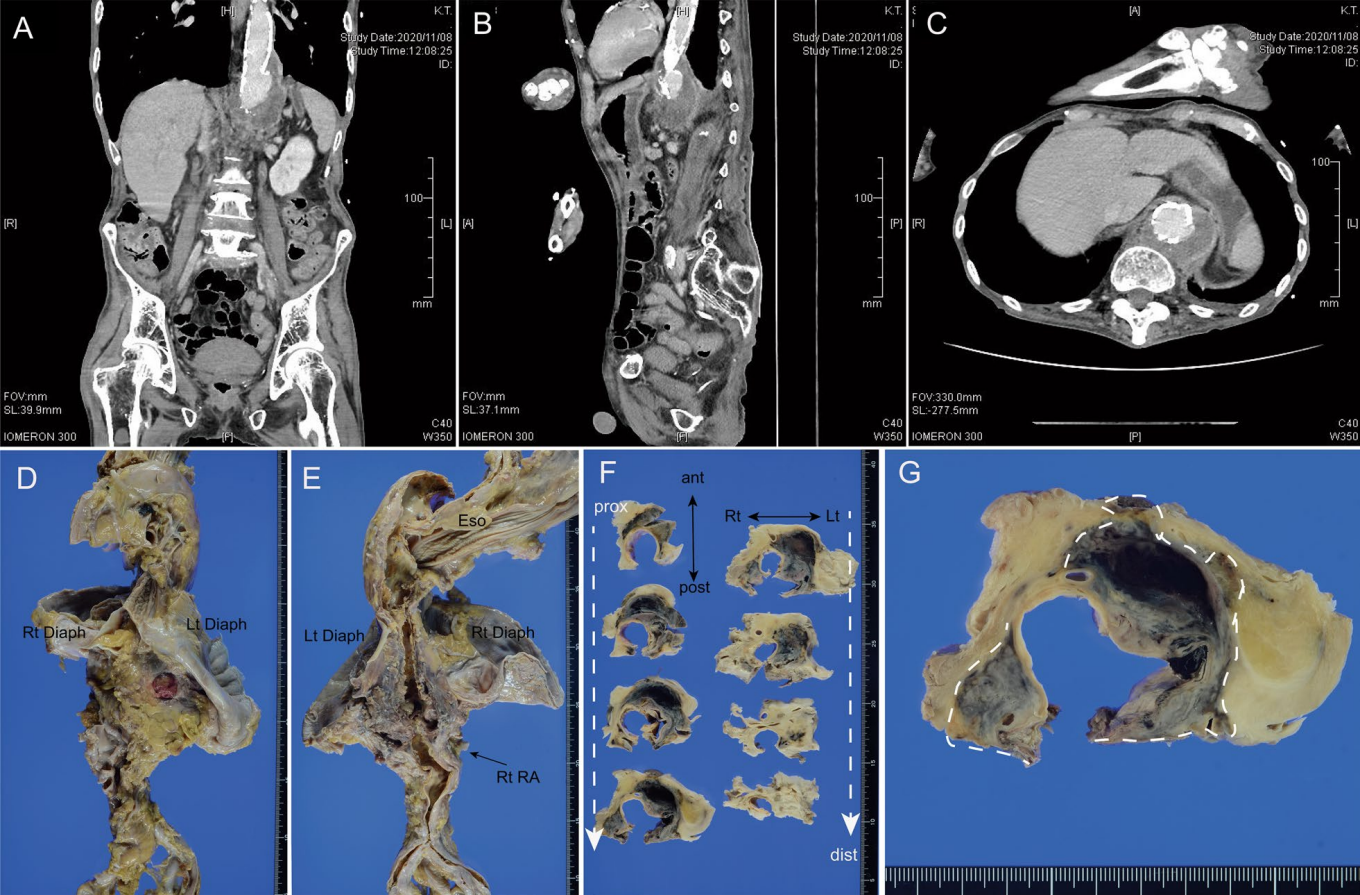
Radiological and macroscopic appearance of the patient in this case report. **A**–**C** Computed tomography scan of the abdomen and pelvis at admission. **A** Coronal section, **B** sagittal section, **C** axial section. **D**–**G** Macroscopic appearance of the infective aortic aneurysm. **D**, **E** Anterior (**D**) and posterior (**E**) views of the aorta and circumferential structure. Dilatation of the suprarenal aorta can be seen. *Rt Diaph* right diaphragm, *Lt diaphragm* left diaphragm, *Eso* esophagus, *Rt RA* right renal artery. **F**, **G** Transverse section of the suprarenal aorta. Partial loss of the aortic structure and saccular outpouching of the aneurysmal wall. Dotted line shows the outline of the aneurysmal wall. *Prox* proximal, *Dist* distal, *Ant* anterior, *Post* posterior, *Rt* right, *Lt* left

His vital signs were as follows: blood pressure, 120/69 mmHg; heart rate, 90 beats per minute; and body temperature, 36.5 °C. The Numerical Rating Scale pain score (maximum points: 10) was 5 [4]. Clinical examination showed right-sided cervical lymphadenopathy and percussion tenderness of the bilateral costovertebral angle and spinal region between Th12 and L2. The results of the laboratory investigations are shown in Table 1. Marked leukocytosis (17,900/µL) with neutrophilia and a high C-reactive protein level (7.43) were observed. Contrast-enhanced computed tomography showed a suprarenal saccular aneurysm (8 cm longitudinally and 5 cm transversely) with enhancement of periaortic soft tissue (Fig. 1A–C). The attending physician strongly suspected IAA as the clinical diagnosis, and the patient was given ampicillin–sulbactam.

**Table 1:** Laboratory findings at the time of admission

Blood count (normal range)		
White blood cells	17,900	(3.3–8.6 × 10^3^)/µL
Red	3.25 × 10^6^	(4.35–5.55 × 10^6^)/µL
Hemoglobin	11.5	(13.7–16.8) g/dL
Hematocrit	32.1	(40.7–50.1) %
Platelet	130	(158–348 × 10^3^)/µL
Biochemistry (normal range)		
Aspartate transaminase	22	(13–30) IU/L
Alanine transaminase	14	(10–42) IU/L
Lactate dehydrogenase	140	(124–222) IU/L
Alkaline phosphatase	98	(38–113) IU/L
r-GTP	28	(13–64) IU/L
Creatinine kinase	35	(59–248) IU/L
Total bilirubin	1.9	(0.4–1.5) mg/dL
Amylase	23	(44–132) IU/L
Total protein	6.5	(6.6–8.1) g/dL
Albumin	4.4	(3.8–5.2) g/dL
Urea nitrogen	18.2	(8.0–20.0) mg/dL
Creatine	0.57	(0.6 < 1.2) mg/dL
eGFR	112	(> 90)
Sodium	132	(138–145) mmol/L
Potassium	3.8	(3.6–4.8) mmol/L
Chloride	100	(98–109) mmol/L
Calcium	9.4	(8.8–10.1) mg/dL
Total cholesterol	125	(142–248) mg/dL
Triglyceride	39	(30–149) mg/dL
C-reactive protein	7.43	(< 0.14) mg/L
Glucose	217	(73–109) mg/dL
D-dimer	11.3	(< 0.5 mg/mL)
Arterial blood gas analysis (normal range)		
pH	7.524	(7.350–7.450)
pCO_2_	33.1	(35–48) mmHg
pO_2_	62.4	(83–108) mmHg
HCO_3_	27.1	(22.0–26.0) mmol/L
ABE	4.7	(− 2.0–2.9) mmol/L
Peripheral blood finding (normal range)		
Neutrophil	95.4	(45.0–73.4)%
Eosinophil	0.0	(1.0–5.0)%
Basophil	0.0	(0.0–3.0) %
Lymphocyte	2.5	(25–45) %
Monocyte	2.1	(3.0–10.0) %
Prothrombin time	14.2	(9.5-12) seconds

*g-GTP* g-glutamyl transpeptidase, *eGFR* estimated glomerular filtration rate, *ABE* actual base excess

The blood culture demonstrated *P. multocida*. The patient was reinterviewed, which revealed several histories of animal bites by a domestic cat. Additional radiological investigation 2 and 3 days after admission showed that the size of the aneurysm had increased. The physician and surgeon evaluated the IAA of the patient to be at a high risk of rupture and considered to administer surgical intervention; however, the physician and surgeon suspended the operation after discussion with the patient and his family members because of poor general condition. Although the antibiotic therapy was changed to meropenem, his general condition did not improve by treatment, and the patient died 16 days after admission.

**Fig. 2:**
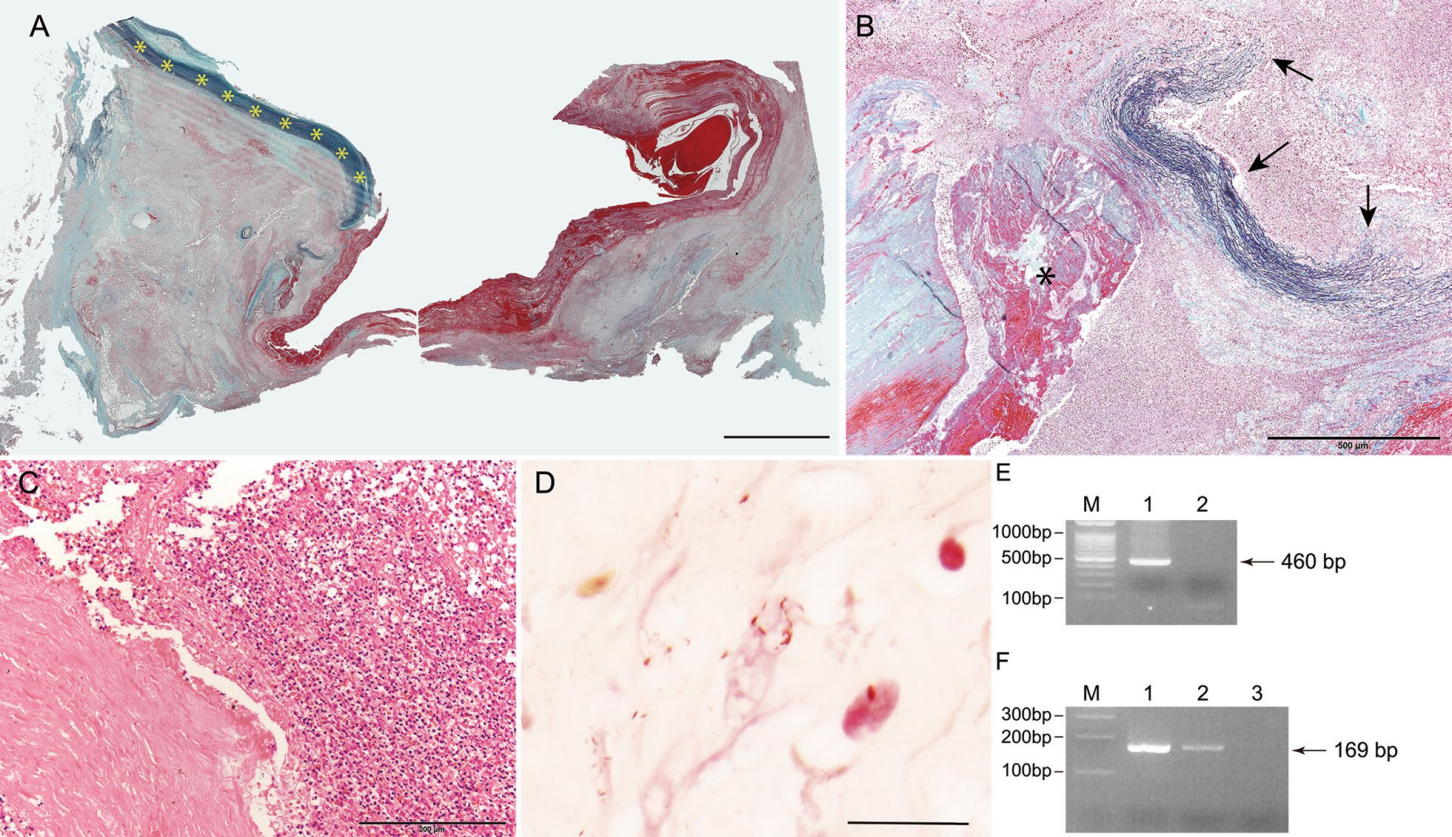
Microscopic appearance of the patient in this case report. **A**, **B** Elastica-Masson staining, **C** hematoxylin–eosin staining, **D** Brown–Brenn Gram staining. **A** Low-power view of the aneurysm (margin between the native aorta and aneurysmal lesion). Loss of the aortic intima and media in the area of the aneurysm. Yellow asterisks showed the media of the native aorta. **B** Fragmented aortic media (arrows) and atheromatous plaque (asterisk) in the aneurysmal wall. **C** Marked infiltration of neutrophils in the aneurysmal wall. **D** Bacilli stained red was detected by Brown–Brenn Gram staining. **E** Multiple capsular PCR typing system for *P. multocida* isolated. The isolate only showed the expected PCR product of approximately 460 bp but negative for types A, B, D, E, and F. The PCR products were run by a 1.5% (w/v) agarose gel electrophoresis in a 1× TBE Buffer system and stained with ethidium bromide. M; marker, Lane 1: *P. multocida* isolated, Lane 2: PCR-negative control. **F** The PCR reaction assay for identifying *P. multocida* in the aorta of the case. Specific targets were amplified by PCR using the *kmt1* gene universal primers, showing detection of the 169-bp products. The products were electrophoresed on a 3% agarose gel. M; marker, Lane 1: the positive control, Lane 2: the case, Lane 3: PCR-negative control. Scale bar = 2 mm (**A**), 500 µm (**B**), 200 µm (**C**), and 10 µm (**D**)

Autopsy was performed at 1 and half hours after the death and revealed that the suprarenal abdominal area was dilated and the lower esophagus was compressed. Horizontal section showed partial loss of the aortic wall in the suprarenal abdominal aorta. Degenerative tissues, including blood coagula, a finding consistent with saccular outpouching from the aortic wall, were continuously observed in the area with loss of the aortic wall (Fig. 1D–F). No hemorrhage was found in the abdominal cavity. Microscopically, in the area where macroscopic loss of the aortic wall showed loss of the native aortic wall, the intima and media were fragmented or completely replaced by degenerative tissue and blood with marked infiltration of neutrophils (Fig. 2A–C). Therefore, the aortic lesion was consistent with mycotic false aneurysm or pseudoaneurysm. The size of the maximal outpouching area was 3 cm in diameter. Atherosclerosis of the aorta was severe, and calcified foci were found around the base of the pseudoaneurysm. Brown–Brenn Gram staining detect red stained Gram-negative cocci in the aneurysmal wall, although Gram and Grocott staining were negative (Fig. 2D).

The QIAamp DNA Mini Kit and the GeneRead DNA FFPE Kit (QIAGEN, Hilden, Germany) were used for the genomic extraction from colonies of *P. multocida* and formalin-fixed paraffin-embedded (FFPE) specimens of IAA, respectively, based on the manufacturer’s instructions and a previous study [5].

The multiplex polymerase chain reaction (PCR) method was applied to the extracted DNA from colonies of *P. multocida* isolated, producing amplification using universal primers for targeting the *kmt1* gene and three serogroup-specific primers for determining *P. multocida* serogroups A, B, D, E, and F [6]. The resultant multiplex PCR of *P. multocida* isolates showed the expected PCR product (approximately 460 bp) with *P. multocida-*specific universal primers (Fig. 2E). The tested isolates were negative for types A, B, D, E, and F. Then, we employed 16S rRNA gene sequencing [7]. The nucleotide sequence of the amplified fragments was ascertained within the GenBank database using the basic local alignment search tool; *P. multocida*, which is composed of three subspecies, was the best sequence match at 100%. For the molecular detection of *P. multocida* in the FFPE specimen, a PCR assay was performed to amplify short fragments using a new universal reverse primer for the *kmt1* gene (5′-TCT GCC CAA CAA AAC TGT GCT TTT C-3′). The extracted DNA from the isolated colonies of *P. multocida* was also prepared as positive controls. The results revealed that the *kmt1* gene specific for *P. multocida* was amplified in the DNA extracted from the FFPE specimen (Fig. 2F).

As other pathological findings, squamous cell carcinoma of the larynx (1.5 cm in diameter, pT3, pN2a, pStage IVa), alcoholic liver cirrhosis, diabetic nephrosclerosis, and acute cholecystitis were found. The cause of death was judged as sepsis due to infection with *P. multocida*.

To the best of our knowledge, four cases with IAA of the native aorta with *P. multocida* infection have been reported (Table 2) [8–11]. All five cases, including the patient in this case report, were male and had a history of animal contacts, such as a bite or scratch. Of these cases, some had aneurysms developing in the thoracic and abdominal aorta, and two cases, including the patient in this case report, demonstrated a pseudoaneurysm. Four of the five cases had a possible risk factor for infection, including collagen disease (rheumatoid arthritis), alcohol addiction, liver dysfunction, and diabetes mellitus. Furthermore, seven cases of aortic endograft infection have been reported (Additional file 1: Table S1). All cases had a history of animal bites, but four of seven cases did not have these possible risk factors.

**Table 2:** Infective aortic aneurysm in the English literature

	Age (y.o)	Sex	Symptoms	Contact with animal	Possible risk factor	Type of aneurysm	Location	Diameter (cm)	Treatment	Outcome	Ref.
1	61	M	Fever, abdominal mass	+	Rheumatoid arthritis	NS	Infrarenal	NS	Operation due to rupture	Intraoperative death	[8]
2	64	M	Fever, abdominal pain	+	Alcohol addiction	NS	Infrarenal	8	Operation and antibiotics	Recovery	[9]
3	54	M	Fever, mental confusion	+	Liver cirrhosis	NS	Thoracic and abdominal (3 lesion)	< 3 cm	Antibiotics	Recovery	[10]
4	61	M	General malaise	+	None	Pseudo	Thoracic	NS	Operation and antibiotics	Recovery	[11]
This case	76	M	Fever, consciousness disturbance	+	Alcohol addiction, DM, liver cirrhosis	Pseudo	Suprarenal	3	Antibiotics	Death due to sepsis	

*Ref.* references, *M* male, *NS* not shown

## Discussion

Saccular outpouching of the aneurysmal wall that was associated with transmural acute inflammation containing numerous neutrophils was the typical pathological appearance of IAA [12]. Lesions typically arise in areas of preexisting vascular dilation (e.g., due to systemic arterial hypertension) or injury (e.g., surgical or invasive monitoring) or in a preexisting atheromatous plaque [13]. Many patients with IAA undergo open surgical repair or endovascular aortic repair because of the high prevalence of rupture [1, 2],. In the patient in this case report, rapid progressive worse general condition inhibited radical treatment. Although the number of cases was limited, rupture of IAA and/or sepsis development may be associated with a poor prognosis.

Many *Pasteurella* species are opportunistic pathogens [3]. Zoonotic transmission of *P. multocida* to humans usually occurs through animal bites or contact with nasal secretions. Although *P. multocida* infection most commonly presents as localized infections, such as cellulitis or abscesses, cases with aortitis, bacteremia, meningitis, respiratory complications, and peritonitis with *P. multocida* infection have been reported [3]. Such a severe infection usually causes immunosuppression, and most cases have a history of liver cirrhosis, solid tumors, and hematological malignancies [14]. Our literature review revealed that rheumatoid arthritis, liver disorder, alcohol addiction, and diabetes mellitus may increase the risk of IAA with *P. multocida* infection. A large observational study of patients with rheumatoid arthritis revealed that individuals with rheumatoid arthritis are at an increased risk of hospitalized infection compared with those without rheumatoid arthritis [15]. Immune dysfunction seen in liver cirrhosis progression may significantly affect the susceptibility of the host and may increase the risk of developing uncommon infection presentation, as the bacteremia found in the patient in this case report [16]. Furthermore, patients with diabetes mellitus have a 4.4-fold greater risk of blood stream infection and are more vulnerable to sepsis of uncertain cause than those without diabetes mellitus [17]. Alcohol consumption can promote organ inflammation, which has consequences on host immunity, and antigen presentation function is negatively affected in patients with chronic alcohol addiction, thereby reducing the host’s defense [18]. Accordingly, the patient in this case report was a high-risk participant for developing severe *P. multocida* infection. Conversely, our literature review revealed that endograft infection with *P. multocida* may frequently occur without immunocompromised state, compared with IAA cases in the native aorta.

This is the first case report in the literature to demonstrate an amplified *P. multocida* in the DNA from the FFPE specimen of IAA. Blood culture is the best method and the first choice of organism identification in cases with hematogeneous pathogen infection. Conversely, one of the significances of PCR using an FFPE specimen is the demonstration of local infection to various organs, including the aorta. Although IAA in most participants with is considered to occur due to hematogeneous pathogen infection, which infect the endothelium, according to two systemic literature reviews on IAA, 23.3% and 29.7% were culture-negative [1, 2]. This case report also demonstrated the use of genetic investigation using FFPE for detecting *P. multocida* in culture-negative IAA cases with a history of various types of contact, especially bites. Invading pathogens both in saliva and uncleaned hand or other skin surfaces may increase the risk of *P. multocida* infection. Although the characteristics of patients with IAA were not significantly different among infected bacterial subgroups, the identification of bacterial species is essential for deciding the therapeutic strategy, particularly antibiotics selection.

This case report showed the clinicopathological appearance of IAA and sepsis due to *P. multocida* infection. The perioperative mortality rate of infectious aortic aneurysms is 15–20%, with worse outcomes in the setting of Gram-negative organism infections and/or ruptures [13]. Early detection and treatment may be essential for IAA and/or sepsis with *P. multocida* infection because many patients with IAA of the native aorta have an immunocompromised state.

## Conclusion

This case report, including a literature review, revealed that careful follow-up may be significant for the early detection of *P. multocida* infection when the participant with immunocompromised background due to liver disorder, alcohol addiction, diabetes mellitus, and/or history of aortic surgery was harmed, especially was bitten by an animal, in addition to its clear clinicopathological appearance.

## Supplementary Information

**Additional file 1: Table S1.** Aortic endograft infection due to *Pasteurella multocida* in the English literature.

## Data Availability

Upon request from the corresponding author.
