# Supplementary material for: An autopsy case of infective aortic aneurysm with Pasteurella multocida infection: clinicopathological appearance and a review of literatures
Source: Ann Clin Microbiol Antimicrob. 2023 Jul 11;22:58. doi: 10.1186/s12941-023-00611-0 (PMC10334504; doi:10.1186/s12941-023-00611-0)
Supplement: Supplementary file 1 — Additional file 1: Table S1. Aortic endograft infection due to Pasteurella multocida in the English literature. [file 12941_2023_611_MOESM1_ESM.docx]

Table S1. Aortic endograft infection due to *Pasteurella multocida* in the English literature

|  | Age (y.o) | Sex | Symptoms | Contact with animal | Possible risk factor | Location | Treatment | Outcome | Ref |
| --- | --- | --- | --- | --- | --- | --- | --- | --- | --- |
| 1 | NS | NS | Fatigue, weight loss, back pain | + | malnourishment | Infrarenal | Percutaneous drainage and antibiotics | recovery | 1 |
| 2 | 61 | M | Malaise, fever, chills and groin pain | + | None | Aortofemoral bypass | Operation and antibiotics | recovery | 2 |
| 3 | 66 | M | Fever, mental confusion | + | Liver cirrhosis | Infrarenal | Antibiotics | recovery | 3 |
| 4 | 78 | F | Drainage from the right femoral incision | + | None | Infrarenal | Operation and antibiotics | recovery | 4 |
| 5 | 69 | F | Fever, hematuria, abdominal pain, flank pain. | + | None | Infrarenal | Operation and antibiotics | recovery | 5 |
| 6 | 58 | M | Abdominal and back pain | + | HIV infection | Infrarenal | Operation and antibiotics | recovery | 6 |
| 7 | 68 | M | Fever, cellulitis | + | None | Infrarenal | Operation and antibiotics | recovery | 7 |

Ref, references; NS, not shown, M, male; F, female.

References

1. Jayakrishnan TT, Keyashian B, Amene J et al. Aortic endograft infectuin by Pasteurella multocida : a rare case. Vasc Endovascular Surg, 2016; 50: 435-437.
2. Kalish SB, Sands ML. Pasteurella multocida infection of a prosthetic vascular graft. JAMA 1983; 249: 514-515.
3. Robbins A, Fouilhé L, Job L, et al. Concomitant *Pasteurella multocida* aortic endograft infection and *Bartonella* *henselae* endocarditis. Med Mal Infect 2015; 45: 424-426.
4. Sannella NA, Tavano P, McGoldrick DM, et al. Aortic graft sepsis caused by *Pasteurella multocida*. J Vasc Surg 1987; 5: 887-888.
5. Shalan A, Wilson N, Poels J, et al. The case of the neighbour’s cat causing a symptomatic (mycotic) aortic aneurysm and an infected graft. EJVES Short Rep 2017; 37: 18-21.
6. Teso D, Williams S, Karmy-Jones R. *Pasteurella multocida* infection of an abdominal aortic endograft. World J Radiol 2013; 5: 17-19.
7. Silberfein EJ, Lin PH, Bush RL, et al. Aortic endograft infection due to *Pasteurella multocida* following a rabbit bite. J Vasc Surg 2006; 43:393-398.
